# Population genetics of 30 insertion/deletion polymorphisms in the Bahraini population

**DOI:** 10.1038/s41598-021-86386-w

**Published:** 2021-03-25

**Authors:** Noora R. Al-Snan, Sabah Shabbir, Sahar S. Baksh, Mashael AlQerainees, Mahdi Haidar, Safia A. Messaoudi, Moiz Bakhiet

**Affiliations:** 1 Forensic Science Laboratory, Directorate of Forensic Science, General Directorate of Criminal Investigation and Forensic Science, Ministry of Interior, Manama, Kingdom of Bahrain; 2 Centre for Forensic Science, Department of Pure and Applied Chemistry, University of Strathclyde, Glasgow, Scotland, UK; 3 Kuwait Identification DNA Laboratory (KIDL), General Department of Criminal Evidence, Ministry of Interior, Kuwait City, Kuwait; 4 Forensic Sciences Department, College of Criminal Justice, Naif Arab University for Security Sciences, Riyadh, Saudi Arabia; 5 Department of Molecular Medicine, College of Medical and Medicine Sciences, Arabian Gulf University, Manama, Kingdom of Bahrain

**Keywords:** Genetic association study, Genetics

## Abstract

This paper evaluates the forensic utility of 30 insertion-deletion polymorphism (indel) markers in a sample from the Bahraini population using the Qiagen Investigator DIPplex Kit. Allele frequencies and forensic stats of the 30 indels were investigated in 293 unrelated individuals from different governorates of the Kingdom of Bahrain. None of the markers showed significant deviation from Hardy Weinberg equilibrium except for HLD88 locus and no linkage disequilibrium were detected between all possible pair of the indel loci, assuming that these markers are independent and their allele frequencies can be used to calculate the match probabilities in the Bahraini population. The high power of discrimination (CPD = 0.9999999999998110) and the low combined match probability (CPM = 1.89 × 10^−13^) indicate that these markers are informative and can be successfully used for human identification in terms of forensics and paternity. Genetic distances and relatedness were displayed through multidimensional plotting and phylogenetic tree using various populations in the region. Our study showed that the Bahraini population was clustered with neighboring countries such as Kuwait and Emirates which indicates that these closely geographical regions share similar allele frequencies and are more genetically related than other reference population studied.

## Introduction

Insertion and deletion polymorphism which is known as (indel), is a type of genetic variation in which a precise nucleotide sequence is present (insertion) or absent (deletion). Compared to short tandem repeat (STRs), indels are found to give promising results for population genetic studies and forensic identification^1^.

Due to the benefits of their short amplicon lengths and the lack of stutter peaks, these markers can be successfully used to analyze degraded DNA samples in challenging forensic cases^2^. In this study, we have used a commercially available indel kit known as the Investigator DIPplex (QIAGEN, Germany) in a sample from the Bahraini population. This kit contains 30 indel markers, which are located on all chromosomes, and Amelogenin sex-informative marker^3^.

This paper contains population data obtained from 293 unrelated Bahraini individuals from different governorates. Bahrain being in the Persian Gulf, connected to the Eastern Coast of the Arabian Peninsula, Iran, Iraq and Oman^4^. It is one of the most densely populated countries with estimates of Bahrain’s population stood at 1,314,562 persons. Of these, 568,399 are Bahraini citizens (46%) and 666,172 are expatriates (54%)^5^.

Because of the geographic location of Bahrain, the diversity of the population had been affected due to the prehistoric cultural events that took place and the migrations flow in this area^6,7^.

## Materials and methods

### Sample collection

Two hundred and ninety-three (293) buccal swabs were collected using cotton swabs (SceneSafe, UK) from healthy unrelated Bahraini males. The research study was publicized through different media platforms. Participants who wished to contribute their samples communicated with the corresponding author and presented at the General Directorate of Criminal investigation and Forensic Science—Kingdom of Bahrain to deliver their samples for the research after obtaining informed consent.

The age of the participants was ranged from 20 to 70 years old. Ethical review for conducting tests was obtained and approved by the Research and Research Ethics Committee (RREC) (E007-PI-10/17) in the Arabian Gulf University, Manama, Kingdom of Bahrain. All participants agreed to the informed consent which were provided prior to their contribution. All research was performed in accordance with relevant guidelines/regulations. In each case, males declared their ancestry (to the level of paternal grandfather) from four different geographical subdivisions of the country (Capital Governorate, Muharraq Governorate, Northern Governorate and Southern Governorate) were sampled.

### DNA processing

Genomic DNA was extracted using QIAsymphony SP instrument (Qiagen, Germany) following magnetic beads principal. Subsequently, the extracted DNA was quantified using Quantifiler HP DNA Quantification kit (Thermo Fisher Scientific Company, Carlsbad, USA) in the 7500 Real-Time PCR System (Thermo Fisher Scientific Company, Carlsbad, USA) according to manufacturer’s recommendation.

About 0.5 ng of the extracted DNA was amplified using Investigator DIPplex kit (Qiagen, Germany) with full-volume reactions (10.5 µl) following manufacturer’s protocol in 30 cycles conditions via MicroAmp Optical 96-Well Reaction Plate (Thermo Fisher Scientific Company, Carlsbad, USA) along with the provided positive control (9948) and nuclease-free water as a negative control in a Veriti thermal cycler (Thermo Fisher Scientific Company, Carlsbad, USA) following the PCR thermal cycles provided in the DIPplex manufacture protocol.

The PCR products (1 µl) were separated by capillary electrophoresis in an ABI 3500xl Genetic Analyzer (Thermo Fisher Scientific Company, Carlsbad, USA) with reference to the BTO size standard (Qiagen, Germany) in total of 12 µl master mix consisting of BTO size standard and Hi-Di formamide (Thermo Fisher Scientific, Inc., Waltham, MA, USA). GeneMapper ID-X Software v1.4 (Thermo Fisher Scientific, Inc., Waltham, MA, USA) was used for genotype assignment in combination with the Investigator DIPplex Template Files and Qiagen DIPSorter software (Qiagen, Germany). Experiments were performed in the Biology and DNA Forensic Laboratory, Ministry of Interior, Kingdom of Bahrain which is accredited with Collaborative Testing Services (CTS).

### Statistical analysis

Forensic parameters such as match probability (MP), discrimination power (PD), probability of exclusion (PE), polymorphism information content (PIC), number of alleles (Nall) and observed heterozygosity (Ho) and the insertion allele frequencies (+ DIP) and the deletion allele frequencies (-DIP) of the 30 indels were calculated using the STRAF online software (http://cmpg.unibe.ch/shiny/STRAF/)^8^.

Arlequin statistical software v3.5^9^ was used to calculate Hardy–Weinberg equilibrium (HWE) and linkage disequilibrium (LD) tests between all pairs of the 30 indels, and p values were corrected by the Bonferroni^10^.

Interpopulation pairwise genetic distances based on Fst calculated from allele frequencies of the population of Bahrain and the rest of populations extracted from the literature which included Kuwait^11^, UAE ^12^, Iraq^13^, Iran^14^, Turkey^13^, Slovenia^13^, Lithuania^13^, Bangladesh^15^, Indonesia^15^, and Japan^15^ using POPTREE2 software^16^ and represented by a nonmetric multidimensional scaling (NM-MDS) analysis using IBM SPSS Statistics v21.0 Software to investigate the populations structure between Bahraini population and the abovementioned populations based on Fst’s genetic distances.

In order to compare between different genetic structures of the populations, phylogenetic tree was constructed from allele frequency data using the neighbor-joining method^17^ via MEGA X: Molecular Evolutionary Genetics Analysis^18^. The tree was constructed with allele frequency data of thirty indel markers (HLD77, HLD45, HLD131, HLD70, HLD6, HLD111, HLD58, HLD56, HLD118, HLD92, HLD93, HLD99, HLD88, HLD101, HLD67, HLD83, HLD114, HLD48, HLD124, HLD122, HLD125, HLD64, HLD81, HLD136, HLD133, HLD97, HLD40, HLD128, HLD39 and HLD84) for all populations in corrected fixation index (Fst) using neighbor joining for phylogeny in 1000 permutations.

### Ethics approval

Ethical review for conducting tests was obtained and approved by the Research and Research Ethics Committee (RREC) (E007-PI-10/17) in the Arabian Gulf University, Manama, Kingdom of Bahrain.

### Consent to participate

All participants provided informed consent prior to contribution their buccal swab samples.

### Consent for publication

All authors/participants provided consent for publication. All figures are generated from software indicated in the materials and methods.

## Results

### Allele frequencies, forensic parameters and efficiency

Allele frequencies, and forensic efficiency parameters for the 30 indel loci in the Bahraini population are shown in Table 1. The genotypes are available in supplementary material Table 1S.

**Table 1:** Allele frequencies and forensic parameters of 30 loci in 293 Bahrainis.

Marker	N	DIP (−)	DIP (+)	GD	PIC	PM	PD	Hobs	He	PE	TPI	HWE
HLD101	584	0.447	0.553	0.495	0.372	0.376	0.624	0.490	0.451	0.179	0.980	0.006
HLD111	584	0.539	0.461	0.498	0.373	0.373	0.627	0.490	0.492	0.179	0.980	0.550
HLD114	584	0.497	0.503	0.501	0.375	0.362	0.638	0.473	0.477	0.165	0.948	0.178
HLD118	584	0.61	0.39	0.477	0.363	0.406	0.594	0.514	0.432	0.200	1.028	0.278
HLD122	584	0.562	0.438	0.493	0.371	0.347	0.653	0.397	0.492	0.112	0.830	0.154
HLD124	584	0.483	0.517	0.500	0.375	0.360	0.640	0.466	0.498	0.159	0.936	0.815
HLD125	584	0.548	0.452	0.496	0.373	0.398	0.602	0.534	0.466	0.219	1.074	0.259
HLD128	584	0.567	0.433	0.492	0.371	0.352	0.648	0.414	0.440	0.123	0.854	0.593
HLD131	584	0.39	0.61	0.477	0.363	0.374	0.626	0.438	0.477	0.139	0.890	0.215
HLD133	584	0.562	0.438	0.493	0.371	0.360	0.640	0.445	0.501	0.144	0.901	1.000
HLD136	584	0.413	0.587	0.486	0.367	0.369	0.631	0.449	0.501	0.146	0.907	0.294
HLD39	584	0.464	0.536	0.498	0.374	0.364	0.636	0.469	0.486	0.162	0.942	0.282
HLD40	584	0.599	0.401	0.481	0.365	0.385	0.615	0.479	0.491	0.170	0.961	0.235
HLD45	584	0.435	0.565	0.492	0.371	0.371	0.629	0.473	0.495	0.165	0.948	0.905
HLD48	584	0.498	0.502	0.501	0.375	0.356	0.644	0.455	0.465	0.151	0.918	0.008
HLD56	584	0.325	0.675	0.440	0.343	0.407	0.593	0.425	0.485	0.130	0.869	0.629
HLD58	584	0.632	0.368	0.466	0.357	0.384	0.616	0.435	0.501	0.137	0.885	0.352
HLD6	584	0.565	0.435	0.492	0.371	0.402	0.598	0.534	0.501	0.219	1.074	0.129
HLD64	584	0.291	0.709	0.413	0.328	0.430	0.570	0.411	0.500	0.121	0.849	0.242
HLD67	584	0.366	0.634	0.465	0.357	0.374	0.626	0.390	0.493	0.108	0.820	0.001
HLD70	584	0.315	0.685	0.432	0.338	0.409	0.591	0.404	0.496	0.116	0.839	0.197
HLD77	584	0.658	0.342	0.451	0.349	0.386	0.614	0.377	0.413	0.100	0.802	1.000
HLD81	584	0.521	0.479	0.500	0.375	0.366	0.634	0.479	0.500	0.170	0.961	0.481
HLD83	584	0.589	0.411	0.485	0.367	0.391	0.609	0.500	0.486	0.188	1.000	0.226
HLD84	584	0.351	0.649	0.456	0.352	0.379	0.621	0.360	0.493	0.091	0.781	0.097
HLD88	584	0.43	0.57	0.491	0.370	0.366	0.634	0.455	0.474	0.151	0.918	0.000
HLD92	584	0.498	0.502	0.501	0.375	0.377	0.623	0.503	0.481	0.191	1.007	1.000
HLD93	584	0.498	0.502	0.501	0.375	0.361	0.639	0.469	0.492	0.162	0.942	0.008
HLD97	584	0.615	0.385	0.474	0.361	0.360	0.640	0.332	0.498	0.078	0.749	0.351
HLD99	584	0.414	0.586	0.486	0.368	0.369	0.631	0.452	0.456	0.149	0.913	0.001

N, number of samples; DIP(**−**), deletion; DIP(+), insertion; GD, Gene diversity; PIC, Polymorphic information contents; PM, Matching probability; PD, Power of discrimination; Hobs, observed heterozygosity; He, Expected heterozygosity; PE, Power of exclusion; TPI, typical paternity index; HWE, Hardy–Weinberg equilibrium.

There was no deviation from Hardy–Weinberg equilibrium (HWE) after applying the Bonferroni correction value of *P* < 0.00017, except for HLD88 which was still deviated even with the correction. The expected heterozygosities (He) ranged from 0.413 to 0.501 with a mean value of 0.481.The observed heterozygosities (Hobs) ranged from 0.332 (HLD97) to 0.534 (HLD6 and HLD125) with a mean average of 0.450. Values for the polymorphic information contents (PIC) ranged between 0.328 and 0.375.

All markers were highly polymorphic and informative for forensic application using Bahraini population sample. To determine the forensic efficiency, we evaluated power of discrimination (PD), power of exclusion (PE) and matching probability (MP). The combined power of discrimination (CPD) and the combined power of exclusion (CPE) for 30 indel markers were 0.9999999999998110 and 0.99276, respectively. The combined MP was 1.89 × 10^−13^ for Bahrainis, allowing a reliable level of discrimination power in forensic cases. Regarding the allele frequency as indicated with deletion and insertion frequencies shown in Table 1, the deletion frequencies (DIP−) ranged from 0.291 (HLD64) to 0.658 (HLD77) with the mean of above 0.4. Insertion frequencies (DIP+) ranged from 0.342 (HLD77) to 0.709 (HLD64). Linkage disequilibrium tests (*P* < 0.000115 after Bonferroni correction) revealed no allelic association between all possible pairwise combinations of 30 indels, indicating the independence of the 30 indel markers as shown in Table 2S.

### Interpopulation diversity

**Figure 1:**
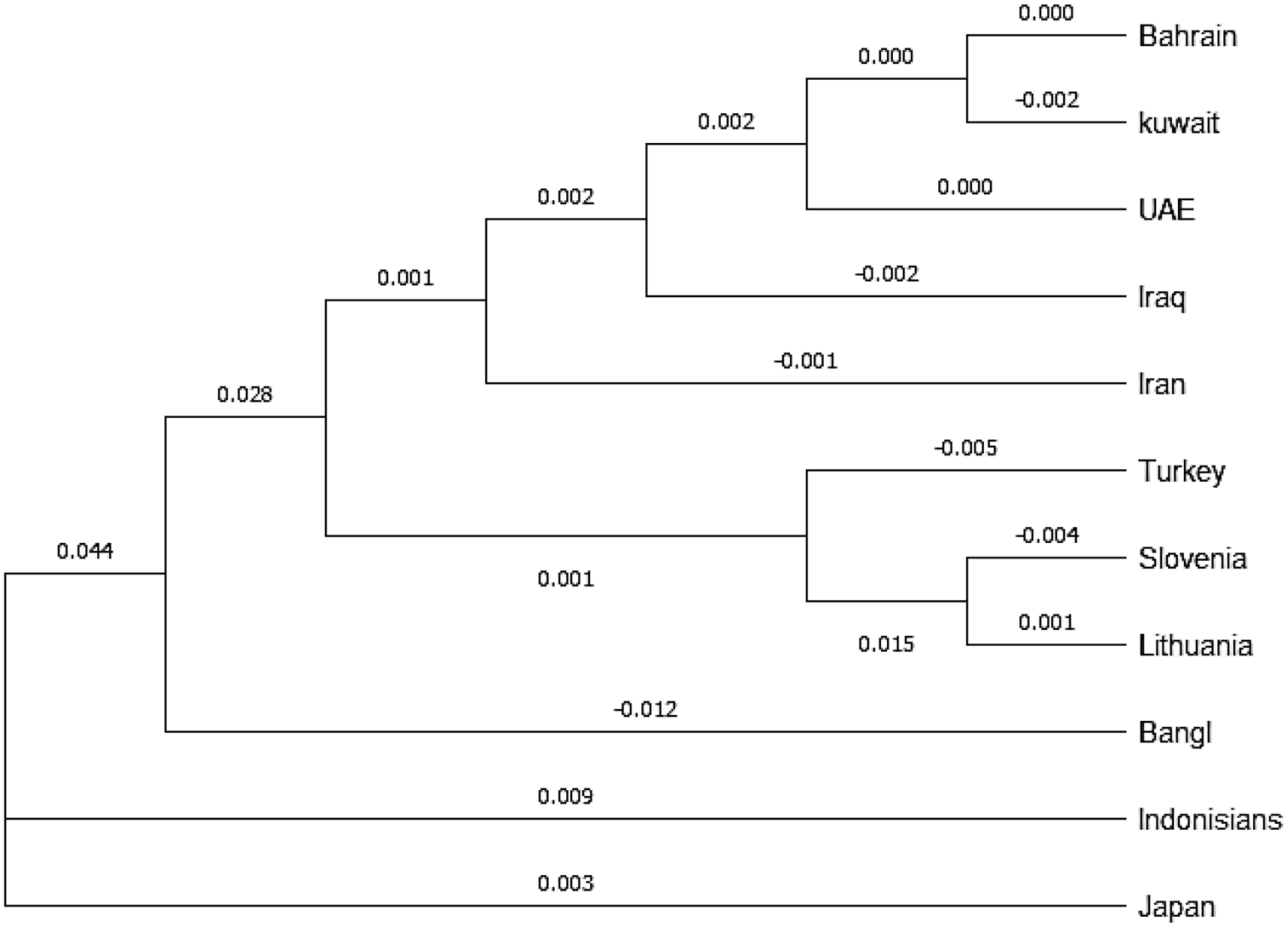
Phylogenetic tree performed using Nei’s DA Distances for the 30 indels estimated among 11 populations.

**Figure 2:**
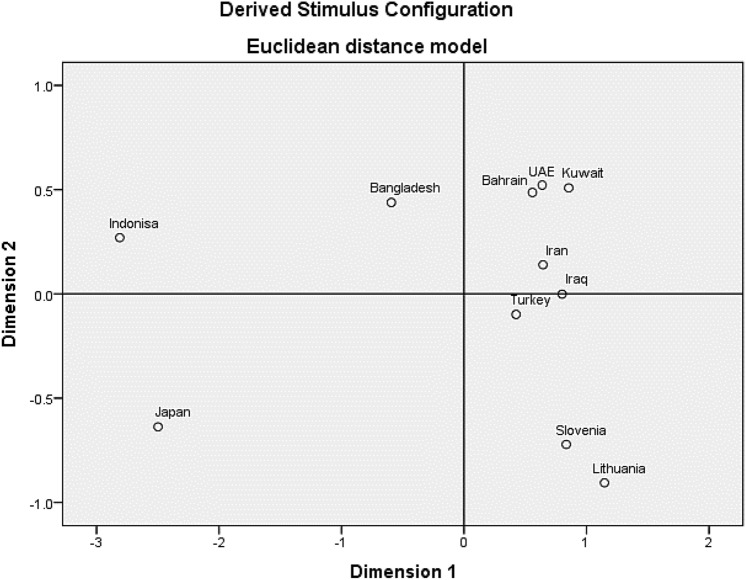
Multidimensional scaling plot (MDS) constructed from pairwise FST distances between 11 populations analyzed with 30 indel markers in the Investigator DIPplex Kit (stress: 0.0252, RSQ: 0.9982) between Bahraini population and other populations.

Determining the genetic structure of populations is becoming increasingly important in genetic studies^19^. To reveal population genetic similarities and divergences between Bahraini population and other populations previously reported, we have constructed the phylogenetic tree (Fig. 1) from allelic frequencies data (deletions and insertions values collected from each marker) by using the neighbor-joining (NJ) method via MEGA X: Molecular Evolutionary Genetics Analysis. Also, by applying the matrix of the Fst genetic distances to generate Multidimensional scale (MDS) plot (Fig. 2).

We have used 10 different populations along with the population of Bahrain: Kuwait^11^, UAE^12^, Iraq^13^, Iran^14^, Turkey^13^, Slovenia^13^, Lithuania^13^, Bangladesh^15^, Indonesia^15^, and Japan^15^. Fst values for allele frequency distribution between Bahraini population and the published groups are shown in (Table 2).

**Table 2:** Nei genetic distance matrix between Bahraini population and other populations showing the Fst values.

	Bahraini	Kuwaiti	Emirati	Iraqi	Iranian	Turkish	Slovenian	Lithuanian	Bengali	Indonesian	Japanese
Bahraini	0										
Kuwaiti	**− **0.003	0									
Emirati	0	**− **0.003	0								
Iraqi	0.001	**− **0.003	0	0							
Iranian	0.004	0.002	0.002	**− **0.003	0						
Turkish	0.002	0	0	**− **0.003	**− **0.003	0					
Slovenian	0.018	0.018	0.02	0.011	0.011	0.006	0				
Lithuanian	0.022	0.021	0.023	0.013	0.015	0.011	**− **0.003	0			
Bengali	0.015	0.021	0.016	0.02	0.013	0.014	0.031	0.038	0		
Indonesian	0.083	0.091	0.085	0.089	0.083	0.071	0.092	0.104	0.035	0	
Japanese	0.075	0.085	0.08	0.082	0.077	0.071	0.081	0.09	0.042	0.012	0

It is shown that Bahraini and Kuwaiti populations shared the most genetic relatedness than the other populations, along with the Emirati population. The rest of populations stood distant of genetic association with the Bahraini population. We have also constructed the MDS plot using IBM SPSS Statistics v21.0 Software, and it gave correlating results with the phylogenetic tree.

As Bahraini, Kuwaiti and Emirati populations gave the same clusters in the North East quadrant, while Irani, Iraqi and Turkish in the adjacent south cluster while Slovenian and Lithuanian populations in the South East quadrant. Figure 2 with good accordance to their geographic region.

## Discussion

The forensic utility of 30 insertion-deletions polymorphism (indel) markers in a sample from the Bahraini population was successfully evaluated in this paper using the Qiagen Investigator DIPplex Kit. The deviation of HWE in HLD88 locus could be a result of high diversity of the studied population or due to the high polymorphism of HLD88 locus, which can also be supported by the PD and PM parameters. In earlier studies of autosomal STR^20^, it was indicated that the Bahraini population structure reflected the high level of endogamy, accounting for 20–50% of all marriages compared to other populations in the region^21^. Also, another explanation is the Wahlund effect within the communities; large number of homozygotes due to population substructure^22^.

As for the LD and after applying the Bonferroni’s corrections, it was shown that for all possible combinations located on the same chromosome indicated minor findings for departures from the independence. Therefore, these studied indels in different loci can be counted as independent for calculation of matching probabilities. We have compared Bahraini population data with other populations according to the available data using the accessible loci. Regarding the Interpopulation diversity, the phylogenetic tree was constructed based upon the data from the 11 populations which were consistent with other population data from the region based upon the Fst values obtained.

In order to measure the population differentiation due to genetic structure, Fst values are obtained for different populations. It is shown that the Bahraini population shares comparable results with its neighboring countries (Kuwait and UAE) based on the 30 indel markers which indicates that these population have more genetic flow than other distant population resulting in similar pattern of allele frequency distribution between them. Once more studies of Arab populations in the region become accessible, it may be more probable to develop a greater understanding of the genetic associations between the different populations for the Arabian Peninsula.

This study increases the population database relevant for the application of genetic markers in forensic studies and can be complementary to STRs population genetic studies in many challenging forensic cases. To conclude, this is the first study to report the allele frequencies and forensic statistical parameters of Bahraini population using the 30 insertion and deletion polymorphisms included in the Investigator DIPplex Kit. Interpopulation comparisons showed that differences were high among populations worldwide, which revealed that DIPplex Kit might be performed well in intercontinental forensic population analysis. The 30 indels markers consisting of straightforward genotyping procedure with low mutation rate and high level of information indicates a great potential in forensic investigations especially in cases where degraded or low quality samples gave partial/null profile using the conventional STR markers, or in paternity cases where additional set of markers are needed to increase the power of the evidence.

## Supplementary Information

Supplementary information.

## Data Availability

The datasets generated during and/or analyzed during the current study are available from the corresponding author on reasonable request. Full dataset associated with this article is available in Table 1S and the linkage disequilibrium is available in 2S.

## Code availability

All the software applications are mentioned within the text.
